# Homœopathic Aggravation*Prepared for the Kansas State Homœopathic Society.

**Published:** 1893-08

**Authors:** S. E. Chapman

**Affiliations:** Watsonville, Cal.


### HOMŒOPATHIC AGGRAVATION.*

S. E. CHAPMAN, M. D., WATSONVILLE, CAL.

A false notion concerning the action of homœopathic remedies is prevalent among the laity, and I might add that many practitioners of Homœopathy entertain the same erroneous conception. I refer to the thousands of times expressed opinion that a homœopathic drug could do no harm if it did no good. Several bitter experiences have inculcated the lesson indelibly upon my mind that the above idea is far from being true. When a case has been well taken, it often occurs that the simillimum

---

* Prepared for the Kansas State Homœopathic Society.

is an exceedingly dangerous agent, and will produce disastrous or dangerous aggravations. In some instances this is true of the higher or highest potencies, but I think usually we meet these manifestations from the use of the low or material doses. It is unnecessary to remark that the last observation does not apply to remedies that in the crude state are inert, such as Silica, Lycopodium, Calcarea-carb., etc. Some of the best minds of our school have declared that in tuberculosis pulmonaris, Sulphur, Phosphorus, and a few other of our antipsorics are to be strictly avoided, as they render the case positively incurable, and materially hasten the fatal issue. Whatever the explanation of this fact may be, no homœopath can disregard it without doing disastrous work, both as to the reputation of himself and Homœopathy, and the welfare of his patient. But it is more particularly in the management of everyday acute bedside clinics I desire to offer a few ideas and experiences.

Many years ago I remember attending a young lady through an attack of pneumonia. The disease ran an ordinary course; and, as I saw it fifteen years later, reached convalescence in *spite* of my miserable alternation of two or more remedies. So I made my final visit, and there was not a single symptom upon which to hang a prescription. Convalescence was progressing perfectly and rapidly, and nothing further in the line of medicine should have been given. But he is well advanced in the science of Homœopathy who has learned to let well enough alone. Being so sure that our remedies in potentized form can never do any harm, I left a few powders of Ars.$^{3x}$ to be given every three hours. The result was something terrible. Here was a patient recovering satisfactorily, and what inane devil could have induced me to make this foolish and inexcusable blunder I do not know. In a few hours I was recalled, and witnessed a perfect picture of Arsenical poisoning. The extreme dyspnœa, restlessness, and fear of death, unquenchable thirst, etc., were all present. Hepatization of upper lobe of left lung soon developed. (The lower lobe of right lung was the seat of the original attack.) I had now to deal with one of the worst cases of pneumonia that I have ever seen recover, all due to three or four powders of Ars.$^{3x}$.

I learned a lesson from this fearful mistake that I have never forgotten. I have often tried to analyze my motive for giving Ars. in this case. Reason I certainly had none, and I remember mumbling some sort of nonsense about "tonic effect." But I think the uppermost thought in my mind was that something was expected of me by the friends, and it did not make any difference what homœopathic remedy I gave, as it could not possibly do any harm.

I have now to relate one of the most painful experiences of my professional life. It is one of the memories I fain would forget, and I only drag it to light in the hope that it may stand as a danger mark to others as it has since done to myself.

A teething babe of fifteen months came into my hands for treatment. Chamomilla was indicated, and under that remedy there was temporary improvement. But three or four days later I was called in again, and found the following symptoms:

Frequent diarrhœic discharges.

Stools green as grass.

Colic before and during stool.

Constant nausea.

Skin moist and pallid.

Hands cold as death.

Great lassitude and desire to remain quiet.

This is an Ipecac picture that the tyro in homœopathics should recognize. My intention was to have given this remedy in the 30x or 200x, and I assured the parents that there was no occasion for alarm. Turning to my medicine case I found that by some strange and sad misfortune I had but the 2x trit. With an ill-defined sense of disappointment—which I now wish had been intensified ten thousand times—I dissolved two grains of the accursed stuff in half a glass of water, and ordered a teaspoonful to be given every half-hour until improvement was apparent. In less than an hour I was hastily summoned, and found terrible exacerbation of all symptoms, particularly that of nausea. The second dose had been given and the nausea was agonizing and incessant. Suffice it to say that in a few moments after my return—it died! By the light of a later and better experience I see that a drop or pellet of Ipecac$^{200x}$ would have saved this precious life; and I can only offer St. Paul's excuse for killing God's saints: "I did it ignorantly in unbelief."

But a short time after this most painful experience I met with another that was fully as instructive, and infinitely pleasanter in its termination. The patient was a lady who had been an invalid many years, and had taken enormous quantities of crude drugs from allopathic hands. This had continued until her stomach revolted and would tolerate no more massive dosage. And this extremity was Homœopathy's opportunity. I will give as concise a picture of the case as possible. A diagnosis I will not offer as the prescription was based entirely upon symptomatology.

A large, fleshy, fair woman, aged fifty years.

Sitting perpendicularly in bed; could not bend forward because of enormous gaseous distention of bowels and stomach. Lying back the slightest degree impossible because of dyspnœa.

Very loud and nearly constant belching that afforded no relief.

Tremendous palpitation of the heart that shook the bed.

Several severe attacks of hemoptysis during the day, due undoubtedly to mitral insufficiency.

A cold, general perspiration, very profuse. During my examination a peculiar and prominent symptom developed: The feet became very uneasy, and finally the heels pounded the bed with great rapidity like a pair of drumsticks. This last symptom was a puzzler, and I could remember nothing like it under any remedy. Zincum-met. occurred to my mind as having excessive fidgetiness of the feet, but that remedy could not cover the other prominent indications. So leaving the pedals to take care of themselves, I dropped one minim of China$^{30x}$ upon her tongue. In two or three minutes her eyes flew open, stared me in the face, and she cried out: "My God! doctor, what have you given me? I'm drunk!" She fell back upon her pillow with these words, and was immediately asleep. The friends were frightened and astounded, but I assured them that she was all right. As I looked upon this magical effect of the infinitesimal dose, *I was born into the kingdom!* The damnable veils and fogs of materialistic sophistries and unbelief were torn aside forever, and I stand to-day a champion for the Homœopathy of Hahnemann.

But to return to my patient. She slept soundly all night, a thing that she had not done for many months. Two or three more doses of the remedy were given as required, and in a week she was walking about her garden.

The point I would accentuate in this report is this: Had I given her a material dose of China, it would have killed as certainly as did Ipecac$^{2x}$ the poor babe.

My honest and earnest advice to all hearers or readers of this paper who are not sound in the faith and practice of Homœopathy is that you immediately abandon all slovenly prescribing, and diligently seek the indicated remedy at all times. It is truly wonderful how satisfactory and beloved your work becomes, and it is a sure road to professional and financial success.

I am not making a fight for high potency. The man who seeks and finds the simillimum will learn to dread it in material doses as he would a deadly serpent.

